# The WID‐qEC test: Performance in a hospital‐based cohort and feasibility to detect endometrial and cervical cancers

**DOI:** 10.1002/ijc.34275

**Published:** 2022-10-01

**Authors:** Lena Schreiberhuber, Chiara Herzog, Charlotte D. Vavourakis, Elisa Redl, Christine Kastner, Allison Jones, Iona Evans, Michal Zikan, David Cibula, Peter Widschwendter, Karin Pfau, Barbara Math, Martin Seewald, Sylvain Amory, Peter Obrist, Martin Widschwendter

**Affiliations:** ^1^ European Translational Oncology Prevention and Screening (EUTOPS) Institute Universität Innsbruck Innsbruck Austria; ^2^ Institute for Biomedical Aging Research Universität Innsbruck Innsbruck Austria; ^3^ Department of Women's Cancer UCL EGA Institute for Women's Health, University College London London UK; ^4^ Department of Gynecology and Obstetrics Charles University in Prague, First Faculty of Medicine and Bulovka University Hospital Prague Czech Republic; ^5^ Gynecologic Oncology Center, Department of Obstetrics and Gynecology, First Faculty of Medicine Charles University in Prague, General University Hospital in Prague Prague Czech Republic; ^6^ Department of Gynecology and Obstetrics General Hospital Hall, Tirol Kliniken Hall in Tirol Austria; ^7^ Danube Private University (DPU) Krems an der Donau Austria; ^8^ Tyrolpath Obrist Brunhuber GmbH Zams Austria; ^9^ Department of Women's and Children's Health Karolinska Institutet Stockholm Sweden

**Keywords:** abnormal vaginal bleeding, cervical cancer, DNA methylation, early detection, endometrial cancer

## Abstract

The majority of endometrial and cervical cancers present with abnormal vaginal bleeding but only a small proportion of women suffering from vaginal bleeding actually have such a cancer. A simple, operator‐independent and accurate test to correctly identify women presenting with abnormal bleeding as a consequence of endometrial or cervical cancer is urgently required. We have recently developed and validated the WID‐qEC test, which assesses DNA methylation of *ZSCAN12* and *GYPC* via real‐time PCR, to triage women with symptoms suggestive of endometrial cancer using ThinPrep‐based liquid cytology samples. Here, we investigated whether the WID‐qEC test can additionally identify women with cervical cancer. Moreover, we evaluate the test's applicability in a SurePath‐based hospital‐cohort by comparing its ability to detect endometrial and cervical cancer to cytology. In a set of 23 cervical cancer cases and 28 matched controls the receiver operating characteristic (ROC) area under the curve (AUC) is 0.99 (95% confidence interval [CI]: 0.97‐1.00) with a sensitivity and specificity of 100% and 92.9%, respectively. Amongst the hospital‐cohort (n = 330), the ROC AUC is 0.99 (95% CI: 0.98‐1) with a sensitivity and specificity of 100% and 82.5% for the WID‐qEC test, respectively, and 33.3% and 96.9% for cytology (considering PAP IV/V as positive). Our data suggest that the WID‐qEC test detects both endometrial and cervical cancer with high accuracy.

## Graphical abstract

What's new?

While abnormal vaginal bleeding is a presenting symptom of endometrial and cervical cancers, only a small proportion of women who present with vaginal bleeding have endometrial or cervical cancer. Currently, the tests used to triage women with abnormal bleeding, such as ultrasound or cytology, are subjective and have modest accuracy. Here, the authors demonstrate that a real‐time PCR‐based test, which assesses DNA methylation at three gene regions using a cervical or vaginal sample, is able to identify 100% of women who have cervical or endometrial cancer with a high specificity (>80%), irrespective of the sample collection system.

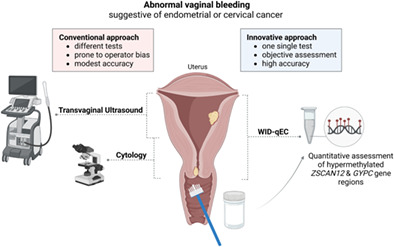

**Abbreviations**
AUC: area under the curve
CAH: complex atypical hyperplasia
CI: confidence interval
CIN: cervical intraepithelial neoplasia
HPV: human papilloma virus
PMR: percentage of fully methylated reference
ROC: receiver operating characteristic

## 1 INTRODUCTION

Endometrial cancer is amongst the cancers with the steepest rise in incidence.^1^, ^2^ Abnormal vaginal bleeding represents the lead symptom of this disease. An estimated 0.33% and 5% to 10% of premenopausal and postmenopausal women with abnormal vaginal bleeding, respectively, are eventually diagnosed with endometrial cancer.^3^, ^4^ In contrast to the second cancer arising from the uterus, that is, cervical cancer, no screening methodology exists for endometrial cancer and cytology shows only modest sensitivity to detect endometrial cancer early.^5^, ^6^ In the past 5 years, global cervical cancer screening coverage has been 32%, ranging between 7% in South‐East Asia and 75% in the European region,^7^ indicating that the majority of global cervical cancer patients may unfortunately not be identified due to screening but may instead present with abnormal vaginal bleeding.

In addition to conventional cytology as the primary test method for cervical screening, the application of liquid‐based cytology, utilizing either ThinPrep or SurePath systems, has been established in many countries with organized cervical cancer screening programs.^8^ Unlike PreservCyt media, the liquid component of ThinPrep, SurePath contains formalin, which crosslinks DNA to proteins and therefore makes DNA‐based tests more challenging.

Recently, we reported the WID‐qEC test, a real‐time PCR‐based test assessing methylated alleles of regions within the *ZSCAN12* and *GYPC* genes.^9^ The WID‐qEC test, based on ThinPrep samples, detects 90% to 100% of endometrial cancers independent of collection devices, menopausal status, age, stage, grade, ethnicity and histology and was able to diagnose 91% of endometrial cancers 1 year prior to diagnosis.^9^

Here, we have employed a case/control and a hospital‐based cohort study to assess whether the WID‐qEC test (a) is also able to detect cervical cancer, (b) is also applicable for SurePath samples utilizing the same threshold as defined in our previous studies, (c) is superior to cytology and (d) retains a low false‐positive rate when assessed within a hospital‐based cohort.

## 2 MATERIALS AND METHODS

**FIGURE 1:**
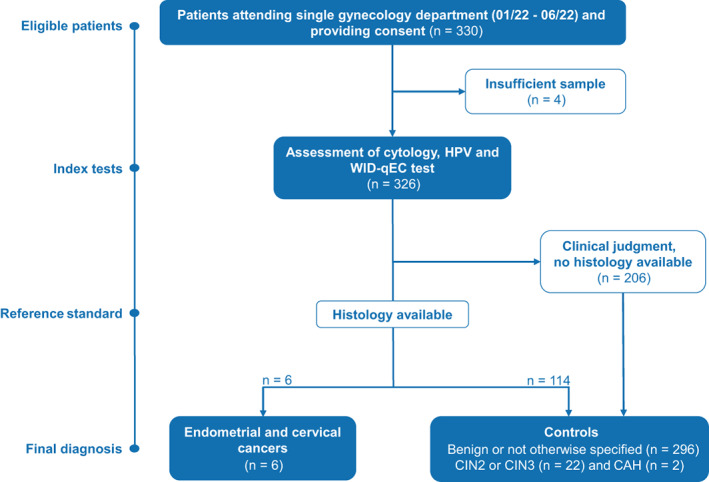
Hospital‐cohort participant flow. CAH, complex atypical hyperplasia; CIN, cervical intraepithelial neoplasia; HPV, human papilloma virus

We have analyzed cervical smear samples from two different settings:

1. *Case/control setting*: Cervical smear samples collected in ThinPrep from women with cervical cancer (n = 23, mean age 50.3 years; 18, 4 and 1 squamous, adeno and small cell cancer, respectively) and matched cancer‐free control women (n = 28, mean age 50.7 years). These samples were collected within the FORECEE study (for details see Refs. [9, 10, 11]). After signing an informed consent, cervical samples were collected at appropriate clinical venues by trained staff and the cervical sample procedures were performed by a small group of research midwives or physicians using the ThinPrep system (Hologic Inc., cat #70098‐002) according to standard operating procedures. Cervical cells were sampled from the cervix using a Cervex brush (Rovers Medical Devices, cat #70671‐001), which was rotated five times through 360° while in contact with the cervix to maximize cell sampling.
2. *Cervical smear samples from a hospital‐based cohort*: Women who attended the gynecology department at the general district hospital in Hall in Tirol were invited to provide a written consent that their sample (collected exactly as described above but in SurePath), surplus to cytological assessment and human papilloma virus (HPV) testing can be utilized for research. A written informed consent was obtained from a total of 330 women. Cytological assessment and HPV testing were carried out in 329 women, of which 326 had sufficient residual sample available for DNA extraction. A total of 6/330 women were eventually diagnosed with a cancer (primary cervical cancers, n = 2; recurrent cervical cancer, n = 1; endometrial cancers, n = 3). The remaining 320/330 were diagnosed with preneoplastic lesions [cervical intraepithelial neoplasia grade 2/3 (CIN2/3), n = 22], had cancers in the past but were currently disease‐free, or had benign conditions (for details see Figure 1). Amongst controls, 34 had previously undergone a total hysterectomy, 25 had a cancer of the vulva, vagina, cervix or uterus, but were free of disease at sample collection, and 23 had previously had a cancer of other organs. Eight patients had prior radiotherapy.

DNA methylation‐specific, quantitative real‐time PCR (MethyLight) analysis was performed as previously described.^8^, ^9^ The final test result (WID‐qEC ∑PMR) is defined as the sum of the percentage of fully methylated reference (PMR) values of the three regions tested (*ZSCAN12* and two regions in *GYPC*). In short, cervical DNA was extracted using the Mag‐Bind Blood and Tissue DNA HDQ 96 Kit (Omega Bio‐tek, cat #M6399‐01) as per the manufacturer's protocol, but with the addition of a heat‐incubation step at 100°C for 40 minutes to ensure formalin‐induced DNA‐protein crosslink‐reversal. DNA was normalized and bisulfite modified using the EZ‐96 DNA Methylation‐Lightning Kit (Zymo Research, cat. #D5033) as per the manufacturer's protocol. Bisulfite modified DNA was amplified using the ×1 Luna Universal Probe qPCR Master Mix (NEB, cat. #M3004G) and primer‐probe sets as described.^9^ All PCR reactions, including the three marker regions in the *ZSCAN12* and *GYPC* (two independent regions) genes as well as the reference gene *COL2A1*, were performed in technical duplicates. PCR reactions were run on the QuantStudio 7 Pro (Applied Biosystems) and results further extracted via the Design and Analysis Software 2.5.0 (Applied Biosystems). The PMR molecules at the target locus were standardized using an R script, dividing the *TARGET*:*COL2A1* input amount ratio (derived using the *COL2A1* standard curve) of a sample by the *TARGET*:*COL2A1* input amount ratio of gBlocks Gene Fragments DNA and multiplying by 100.^9^

All statistical analyses were carried out in R version 4.1.2 (1 November 2021). Receiver operating characteristic curves, areas under the curve and corresponding 95% confidence intervals (CIs) were generated using the pROC package, version 1.18.0. Sensitivity and specificity including 95% CIs were calculated using the bdpv package, version 1.3. Cross tabulation and chi‐square test were performed using the sjPlot package, version 2.8.11.

## 3 RESULTS AND DISCUSSION

**FIGURE 2:**
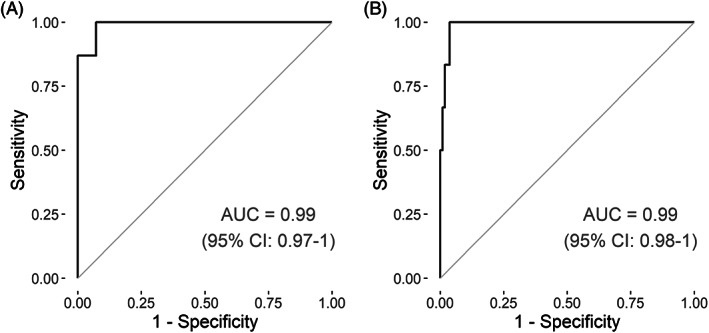
WID‐qEC performance. The WID‐qEC test is assessed in (A) the case‐control set (23 cervical cancer cases and 28 age‐matched control women) and (B) the hospital‐cohort (six cancers—three endometrial and three cervical cancers—320 controls among which 22, two and one were subsequent to sample collection diagnosed with CIN2/3, complex atypical endometrial hyperplasia and highly suspicious endometrium). Receiver operating characteristic curves with the relevant area under the curve (AUC) are displayed. 95% CI, 95% confidence interval

The WID‐qEC receiver operating characteristic (ROC) area under the curve (AUC) to distinguish cervical cancer cases and controls in the case/control setting (n = 51) was 0.99 (95% CI: 0.97‐1.00) (Figure 2A). Applying the same cutoff for the WID‐qEC ∑PMR as previously defined for endometrial cancers (WID‐qEC ∑PMR ≥0.03),^9^ the sensitivity and specificity was 100% and 92.9%, respectively. These data suggested that the WID‐qEC test identified women with cervical cancer with a very high sensitivity and specificity.

Amongst the hospital‐based cohort (n = 330), the ROC AUC distinguishing endometrial or cervical cancers from all controls (including CIN2/CIN3) was 0.99 (95% CI: 0.98‐1; Figure 2B). All six cervical and endometrial cancers were correctly identified (sensitivity 100%, 95% CI: 54.1‐100) using the previously defined threshold. The specificity was 82.5% (95% CI: 77.9‐86.5) when considering all 320 controls and 84.6% (95% CI: 80.0‐88.5) when excluding the 22 CIN2/3 patients. In contrast, the sensitivity of cytology (PAP IV or PAP V) was 33.3% (95% CI: 4.3‐77.7) with a specificity of 96.9% (95% CI: 94.3‐98.5). Considering all cytology results above PAP III as positive, the sensitivity of cytology was 66.7% (95% CI: 22.3‐95.7) with a specificity of 90.9% (95% CI: 87.2‐93.8) and 95.6% (95% CI: 92.7‐97.7) when including or excluding CIN2/3 cases, respectively (Table 1). These data suggest a significantly higher sensitivity of the WID‐qEC test to detect endometrial or cervical cancer cases compared to cytology (difference in sensitivity [≥PAP IV]: 66.6% [95% CI: 23.3‐100]).

**TABLE 1:** Sensitivity and specificity of the WID‐qEC test and cytology to identify women with endometrial and cervical cancer in the hospital‐cohort

	WID‐qEC (∑PMR ≥0.03)	PAP (≥ IV)	PAP (≥ III)
*Including CIN2/3*			
Cases, n	6	6	6
Controls, n	320	320	320
Sensitivity (EC, CC), % (95% CI)	100 (54.1‐100)	33.3 (4.33‐77.7)	66.7 (22.3‐95.7)
Specificity, % (95% CI)	82.5 (77.9‐86.5)	96.9 (94.3‐98.5)	90.9 (87.2‐93.8)
PPV, % (95% CI)	8.1 (5.4‐10.9)	16.5 (5.2‐41.7)	12 (6.6‐20.9)
NPV, % (95% CI)	99.7 (98.5‐99.9)	98.7 (97.8‐99.3)	99.3 (97.9‐99.8)
*Excluding CIN2/3*			
Cases, n	6	6	6
Controls, n	298	298	298
Sensitivity (EC, CC), % (95% CI)	100 (54.1‐100)	33.3 (4.33‐77.7)	66.7 (22.3‐95.7)
Specificity, % (95% CI)	84.6 (80‐88.5)	99.7 (98.1‐100)	95.6 (92.7‐97.7)
PPV, % (95% CI)	9 (5.9‐12.3)	64.8 (16.1‐94.6)	22.1 (11.5‐38.1)
NPV, % (95% CI)	99.7 (98.5‐99.9)	98.8 (97.9‐99.3)	99.4 (98‐99.8)
Assumed population prevalence: 6/330 (1.81%)			

*Note*: We assessed the WID‐qEC test with a predefined threshold (∑PMR ≥0.03) and cytology (using two thresholds) including and excluding CIN2/3 cases within the control group.

Abbreviations: 95% CI, 95% confidence interval; NPV, negative predictive value; PPV, positive predictive value.

Among 56/320 WID‐qEC false positive cases, 10 women were subsequently diagnosed with CIN2 or CIN3, 2 women were subsequently diagnosed with complex atypical hyperplasia (CAH) and 1 woman was suspicious for endometrial cancer (polyp, thickened suspicious endometrium and enlarged lymph node) but refused procedures to obtain a histological diagnosis. HPV infections did not seem to drive false positive WID‐qEC results: Amongst the 298 CIN2/3 negative controls, 1/46 (2.2%) WID‐qEC false positive and 11/252 (4.4%) WID‐qEC true negative women tested positive for HPV16. None of the CIN2/3 negative controls were HPV18 positive and 4/46 (8.7%) WID‐qEC false positive and 26/252 (10.3%) WID‐qEC true negative cases were positive for other oncogenic HPV strains (ie, 31, 33, 35, 39, 45, 51, 52, 56, 58, 59, 66 and 68). In addition, none of the false positive women had previously received radiotherapy.

Amongst the 4/6 cytology false negative cancer cases, three women were subsequently diagnosed with endometrial cancer (two women presented with PAP II, one woman with PAP III), and one woman suffered from an adenocarcinoma of the cervical canal (PAP IIIG).

Our previous findings highlighted that the WID‐qEC detects endometrial cancer across a variety of sampling settings, including self‐sampling. In accordance with these prior findings, our data indicate that this new PCR‐based test is robust and outperforms cytology to detect both cancers arising from the uterus, endometrial and cervical, irrespective of whether the ThinPrep or SurePath system is utilized for liquid‐based cytology sampling. Of note, the ThinPrep system may be the preferred one over SurePath due to the absence of formalin in the PreservCyt media which allows for easier sample processing. Our data also confirm the low sensitivity of cytology to detect endometrial cancer.^5^, ^6^

Overall, our current results, in conjunction with recently published data,^9^ confirm that the WID‐qEC test shows an unprecedented high sensitivity in detecting both endometrial and cervical cancer in symptomatic women irrespective of the collection device, sampling system (ThinPrep or SurePath) or clinical setting. A positive WID‐qEC test in symptomatic women will trigger a comprehensive cervical inspection, colposcopy/biopsy and, especially if the ectocervix does not reveal a cancer, a hysteroscopic assessment along with an endometrial and endocervical curettage in order to obtain tissue for histological assessment.

## AUTHOR CONTRIBUTIONS

**Lena Schreiberhuber:** Conceptualization; Formal analysis; Investigation; Methodology; Writing—original draft; Writing—review and editing. **Chiara Herzog:** Conceptualization; Formal analysis; Writing—review and editing. **Charlotte D. Vavourakis:** Writing—review and editing. **Elisa Redl:** Investigation; Methodology; Writing—review and editing. **Christine Kastner:** Investigation; Methodology; Writing—review and editing. **Allison Jones:** Investigation; Methodology; Writing—review and editing. **Iona Evans:** Investigation; Methodology; Writing—review and editing. **Michal Zikan:** Methodology; Writing—review and editing. **David Cibula:** Methodology; Writing—review and editing. **Peter Widschwendter:** Conceptualization; Methodology; Writing—review and editing. **Karin Pfau:** Methodology; Writing—review and editing. **Barbara Math:** Investigation; Methodology; Writing—review and editing. **Martin Seewald:** Investigation; Methodology; Writing—review and editing. **Sylvain Amory:** Investigation; Methodology; Writing—review and editing. **Peter Obrist:** Conceptualization; Investigation; Methodology; Writing—review and editing. **Martin Widschwendter:** Conceptualization; Formal analysis; Writing—original draft; Writing—review and editing. The work reported in the article has been performed by the authors, unless clearly specified otherwise in the text.

## FUNDING INFORMATION

Our study was funded by the European Union's Horizon 2020 European Research Council Program, H2020 BRCA‐ERC under Grant Agreement No. 742432 as well as the Land Tirol and the charity The Eve Appeal (https://eveappeal.org.uk/).

## CONFLICT OF INTEREST

Chiara Herzog and Martin Widschwendter are shareholders of Sola Diagnostics GmbH. Peter Obrist is a shareholder of Tyrolpath and Tyrolpath is a shareholder of Sola Diagnostics GmbH. Sola Diagnostics GmbH holds an exclusive license to the intellectual property that protects the commercialization of the WID‐qEC test. The other authors have no conflict of interest to declare.

## ETHICS STATEMENT

The cervical cancer case/control study was a sub‐study of the FORECEE (4C) program, which has ethical approval from the UK Health Research Authority (REC 14/LO/1633) and all other contributing centers. The hospital‐based cohort study has received ethical approval from the ethical committee of the Medical University Innsbruck (Ethical Committee Nrs 1389/2020 and 1390/2020 and 1411/2020). All volunteers provided a written informed consent.

## Data Availability

The data that support the findings of our study are available from the corresponding author upon reasonable request.
